# Correlations between Total Antioxidant Capacity, Polyphenol and Fatty Acid Content of Native Grape Seed and Pomace of Four Different Grape Varieties in Hungary

**DOI:** 10.3390/antiox10071101

**Published:** 2021-07-09

**Authors:** Éva Szabó, Tamás Marosvölgyi, Gábor Szilágyi, László Kőrösi, János Schmidt, Kristóf Csepregi, László Márk, Ágnes Bóna

**Affiliations:** 1 Department of Biochemistry and Medical Chemistry, Medical School, University of Pécs, 7624 Pécs, Hungary; szabo.eva.dr@pte.hu (É.S.); gabor.szilagyi@aok.pte.hu (G.S.); janos.schmidt@aok.pte.hu (J.S.); laszlo.mark@aok.pte.hu (L.M.); 2 Department of Pediatrics, Medical School, University of Pécs, 7623 Pécs, Hungary; marosvolgyi.tamas@pte.hu; 3 Institute of Bioanalysis, Medical School, University of Pécs, 7624 Pécs, Hungary; 4 Research Institute for Viticulture and Oenology, University of Pécs, 7634 Pécs, Hungary; korosi.laszlo@pte.hu; 5 Department of Plant Biology, University of Pécs, 7624 Pécs, Hungary; csepregi@gamma.ttk.pte.hu; 6 MTA-PTE Human Reproduction Research Group, 7624 Pécs, Hungary

**Keywords:** grape seed, pomace, antioxidant activity, polyunsaturated fatty acids, polyphenol, resveratrol, rutin, HPLC, GC

## Abstract

Grape pomace is a valuable source of various bioactive compounds such as plant-derived polyphenols and polyunsaturated fatty acids (PUFAs). The commercial demand of grape skin and seed powders as nutraceuticals is still growing. However, no distinction is currently made between unfermented native grape seed and grape seed pomace powders regarding their antioxidant activities. Our aim was to find the relationship between the polyphenol and fatty acid content as well as the antioxidant capacity of native and fermented grape seeds of four different grape varieties harvested in the Villány wine region. According to our results, none of the three investigated polyphenols (resveratrol, rutin, quercetin) could be detected in native grape seed samples in correlation with their significantly lower total antioxidant capacities compared to fermented seed samples. Pinot Noir (PN) grape seed pomace samples with the highest resveratrol and oil content showed significantly higher total antioxidant capacity than Cabernet Sauvignon (CS), Syrah (S) and Blue Portugal (BP) samples. Based on the statistical analysis, positive correlation was found between the fatty acid content and the resveratrol concentration in the pomace samples of different grape varieties. In contrast, rutin concentrations were negatively proportional to the fatty acid content of the fermented samples. No significant correlation was found considering the quercetin content of the samples. According to our findings, grape pomace seems a more promising source in the production of nutraceuticals, since it contains polyphenols in higher concentration and exerts significantly higher antioxidant activity than native grape seeds.

## 1. Introduction

The demand for natural plant-based antioxidants promotes the production of wide varieties of nutraceutical products. Bioactive polyphenols present in the skin and seeds of grapes occur in wine and even in grape pomace after winemaking (fermentation) [1]. Grape pomace, as a relatively inexpensive and abundant source of antioxidants, is commercially available in a form of dietary supplements (powders, tablets, capsules), providing an alternative source of polyphenol intake avoiding wine consumption. Interestingly, no difference is made so far between native grape seed and grape pomace products considering their antioxidant effect. However, the latter seems a more promising raw material in the production of nutraceuticals.

The antioxidant activity of plant polyphenols is well known. These bioactive molecules exert their beneficial effects by neutralizing reactive oxygen species (ROS) and chelating pro-oxidative metal ions [2]. Polyphenols may play a key role in the prevention of degenerative processes, such as cancer [3], diabetes [4], chronic inflammation [5] and aging [6]. In addition, polyphenolic compounds inhibit the oxidation of low-density lipoproteins and prevent platelet aggregation, decreasing the development of cardiovascular and coronary diseases [7]. The level of phenolic compounds in grape seeds depends mostly on the grape variety [8,9,10], ripeness [11,12], growing region [13,14], year of the harvest [8,15,16] and winemaking technology [17].

The oil content of grape seed pomace is about 5–20 wt%, containing linoleic acid (C18:2n-6, LA) and oleic acid (C18:1n-9, OA) as the most abundant fatty acids [18,19,20]. The oil content and the fatty acid composition of grape seeds are affected by ripening [21,22], grape variety [18,19,20,23,24,25,26,27], the year of the harvest [18] and geographical location [28]. The beneficial properties of grape seed oils are associated with polyunsaturated fatty acids (PUFAs). α-Linolenic acid (C18:3n-3, ALA) and LA cannot be synthesized by the human body; they must be ingested with foods. These essential fatty acids decrease the risk of cardiovascular diseases and cancer [18].

Hungary, although a small country in terms of territory, is at the forefront of world wine production: it ranks 14th worldwide and 8th in Europe [29]. In Villány, among other wine-producing regions of Hungary, mainly red wine is produced. Although there are several studies about the oil, fatty acid and polyphenol content as well as the antioxidant capacities of different grape varieties harvested in European countries [30,31,32,33], the amount of Hungarian data is very limited. According to present knowledge, no study has been performed until now that focused on the relationship between fatty acid composition, polyphenol content and antioxidant capacity values of native grape seeds and pomace. Our aim was to find a correlation among these data and to investigate the possible relationship between the concentration of PUFAs and the investigated polyphenols (resveratrol, rutin and quercetin), since they exert beneficial physiological effects.

## 2. Materials and Methods

### 2.1. Chemicals

GC-grade methanol, *n*-hexane and chloroform as well as the ACS grade K_2_CO_3_ were purchased from Merck (Darmstadt, Germany). Puriss. p.a.-grade acetyl chloride and ACS grade pyrogallol were purchased from Sigma-Aldrich (Steinheim, Germany).

Resveratrol, rutin and quercetin standards were obtained from Sigma-Aldrich (Steinheim, Germany). The HPLC-grade acetic acid, ethanol, water and methanol were purchased from Merck (Darmstadt, Germany).

Folin–Ciocalteu (FC) reagent, 2,2′-azino-bis(3-ethylbenzothiazoline-6-sulphonic acid) diammonium salt (ABTS), Trolox (6-hydroxy-2,5,7,8-tetramethylchroman-2-carboxylic acid), horseradish peroxidase, and H_2_O_2_ were purchased from Sigma-Aldrich (Steinheim, Germany). ACS-grade ascorbic acid, Na_2_CO_3_ and FeCl_3_ solutions as well as the HPLC-grade 2,4,6-Tris(2-pyridyl)-s-triazine (TPTZ) and gallic acid were obtained from Sigma-Aldrich (Steinheim, Germany).

### 2.2. Plant Materials

The investigated grape varieties (Pinot Noir, PN; Cabernet Sauvignon, CS; Syrah, S and Blue Portugal, BP) were harvested in 2017 in the Villány wine region (Hungary). The seeds of fermented and unfermented grape berries provided by the Bock winery were removed manually, were washed with distilled water and dried in an exsiccator at 35 °C for 24 h. Dried native and pomace seeds of the four different grape varieties were homogeneously ground by an electric grinder. The samples were stored at −80 °C until further measurements were carried out.

### 2.3. Total Antioxidant Capacity Determination

The antioxidant activities of grape seeds were measured using three different methods. Since individual phenolic compounds may have different responses to different assays, a complex, multi-aspect approach is necessary to obtain a good assessment of the total antioxidant capacities (TACs) of biological samples [34]. Powdered grape seed samples (60 mg) were placed into Eppendorf tubes. For the extraction, 1.5 mL of a 1:1 mixture of ethanol and water was used. Samples were placed into an ultrasonic water bath (Industrial Ultrasonic Cleaner, YS-JP-020, Shenzhen, China) for 15 min at room temperature and then centrifuged (Heraeus Fresco 17 Microcentrifuge, Thermo Fisher Scientific, Bremen, Germany) at 13,000 rpm for 10 min at room temperature. Supernatants were transferred into Eppendorf tubes and pellets were re-extracted following the above procedure two more times. Pooled supernatants of the three repeated extractions made one sample.

The total antioxidant capacities of grape seed extracts were measured by using a modified Folin–Ciocalteu assay [35]. Diluted FC reagent (0.5 mL, 1:10 in distilled water) was mixed with the seed extracts in microplate wells and incubated at room temperature for 5 min. Na_2_CO_3_ solution (0.5 mL, 6 wt%) was added to the extracts and the mixture was incubated for 90 min at room temperature. Absorbance was measured at 765 nm (Multiskan FC plate reader, Thermo Fisher Scientific, Waltham, MA, USA). Calibration was made with gallic acid. To compare the results obtained from the different assays, FC antioxidant capacity values were expressed uniformly as mg ASA equivalents per mg dry grape seed.

Ferric-reducing antioxidant potential (FRAP) is a TAC method based on the reduction of ferric TPTZ to ferrous TPTZ complex. The FRAP reagent was made by mixing acetate buffer (300 mM, pH 3.60), TPTZ solution (10 mM TPTZ in distilled water) and FeCl_3_ solution (20 mM FeCl_3_ in 40 mM HCl) [34]. Seed extracts of 10 µL were incubated with 190 µL of FRAP reagent in microplate wells at room temperature for 30 min (shaking the samples every 10 min), and the absorbance of the samples was measured at 620 nm by using the plate reader. Calibration was made with ascorbic acid. FRAP data of seed extracts were expressed as mg ASA equivalents per mg dry grape seed.

The Trolox equivalent antioxidant activity (TEAC) of samples was measured according to Majer and Hideg [36] with modifications. The blue colored 2,2′-azino-bis(3-ethylbenzothiazoline-6-sulphonic acid) radical cation (ABTS^+^) was prepared in a phosphate buffer (50 mM, pH 6.00), by mixing ABTS^+^ (0.1 mM), horseradish peroxidase (0.0125 µM), and H_2_O_2_ (1 mM). The solution was incubated for 15 min at room temperature. Seed extracts of 10 µL were added to 190 µL of ABTS^+^ solution and the loss of blue color was followed at 651 nm. Calibration was made with Trolox. Total antioxidant capacity values were calculated from TEAC results and expressed as mg ascorbic acid (ASA) equivalents per mg dry grape seed in order to make the results of different assays comparable.

### 2.4. Polyphenol Content Determination by HPLC

The homogeneously grinded grape seed samples (100 mg) were extracted with the 1:1 mixture of ethanol and water (1 mL) for six hours at 80 °C. Extracts were centrifuged at 10,000 rpm for 10 min at room temperature. The supernatants of the samples were filtered through Acrodisc syringe filters (25 mm, 0.2 μm) (Pall Life Sciences, New York, NY, USA) prior to the injection.

The HPLC system used consisted of an Ultimate 3000 SD pump (Dionex Softron GmbH, Germering, Germany), an Ultimate 3000 autosampler, and an Ultimate 3000 RS UV–vis diode array detector. The eluate was monitored at three different wavelengths (306, 350 and 365 nm), respectively, according to the absorption maxima of the investigated polyphenols. Chromatographic separations were achieved using a Kinetex C18 reversed phase column (5 μm, 2.1 mm × 150 mm i.d.) (Phenomenex, Torrance, CA, USA). Chromeleon (Version 6.8, Thermo Fisher Scientific) and Hystar (Version 3.2, Bruker Daltonics, Bremen, Germany) data management software were used to control the equipment and DataAnalysis (Version 4.0, Bruker Daltonics) was used for data evaluation.

A multi-step gradient method was applied, using methanol/water/acetic acid (10/90/1 *v*/*v*/*v*) as solvent A and methanol/water/acetic acid (90/10/1 *v*/*v*/*v*) as solvent B at a flow rate of 0.6 mL/min. The gradient profile was: 0.0–10.0 min, from 0% to 15% B; 10.0–35.0 min, from 15.0% to 35.0% B, 35.0–40.0 min, from 35.0% to 100.0% B followed by a hold of 5 min. The equilibrium time to original conditions was 5 min. The total runtime was 55 min. The injection volume was 5 μL. The column temperature was set to 50 °C. The limit of detection (LOD)/limit of quantification (LOQ) values were 3/10 ng/mL for resveratrol, 6/21 ng/mL for quercetin and 10/34 ng/mL for rutin.

### 2.5. Oil content Determination

The oil content of the different grape seeds was determined in three parallel samples by modified Bligh and Dyer extraction [37,38].

Shortly, for the lipid extraction, 2.5 mL of methanol and 1.25 mL of chloroform, then 1.25 mL of distilled water and 1.25 mL of chloroform, were added to 200.0 mg of powdered grape seed sample. The different phases were separated by centrifugation at 3000 rpm for 15 min at 4 °C. The lower chloroform phase containing the lipids was transferred into a test tube of known weight by using a Brand macro pipette controller. The weight of the N_2_-dried lipids were measured, and the lipid content was calculated. Oil content data are given as g oil in 100 g dried grape seed.

### 2.6. Fatty Acid Analysis

The lipid extract (about 0.1 mg) was transferred into the extraction tube and 2 mL of methanol/hexane (4/1 *v*/*v*) was added. To prevent auto-oxidation, 0.5% pyrogallol was used. During shaking on a vortex mixer, 200 µL of acetyl chloride was added. For derivatization, the reaction tubes were placed into a heating block for 1 h at 100 °C. After cooling down, 4.8 mL of K_2_CO_3_ solution (6% *w*/*v*) was added. The samples were centrifuged at 3200 rpm for 10 min at 4 °C. The upper, fatty acid methyl ester containing hexane phase was transferred to vials and analyzed by gas chromatography (GC).

Fatty acid composition was determined by using Agilent 6890N GC, which consisted of autosampler 7683B, and a flame ionization detector (FID). Separation was performed on capillary column DB-23 (60 m × 0.25 mm × 0.25 µm; Agilent J&W Scientific, Folsom, CA, USA) [39].

The Chromeleon (Version 7.1, Thermo Fisher Scientific, Sunnyvale, CA, USA) data management software was used to control the equipment and for data evaluation.

Peak identification was verified by comparison with standard reference mixtures (NuChek Prep Inc., Elysian, MN, USA: GLC-463, 674, 642 and 643). Fatty acid contents were expressed as weight percent of total fatty acids (wt%).

### 2.7. Statistical Analysis

Statistical analysis was performed with SPSS for Windows 25.0 (SPSS Inc., Chicago, IL, USA); the results of each sample were compared with ANOVA and an independent *T*-test. For correlations between fatty acids, polyphenols and total antioxidant capacity values, Pearson’s correlation was used. The level of significance was *p* < 0.05. All measurements were performed in triplicate and data are presented as mean ± SD.

## 3. Results

### 3.1. Polyphenol Content

The mg/kg concentrations of the three different polyphenols in grape seed pomace were calculated (Table 1). Considering the resveratrol content of the analyzed grape seed pomace samples, the highest concentrations were detected in the PN samples, while BP, CS and S samples contained significantly lower levels of resveratrol. The levels of rutin were significantly higher in BP pomace seeds than in PN and CS samples. The lowest amount of rutin was found in CS samples. S samples showed higher while CS samples significantly lower quercetin content compared to BP and PN pomace seeds with similar quercetin values. Rutin and quercetin values correlated significantly in the investigated samples (Pearson correlation coefficient: +0.645, *p* = 0.024), while there was no significant correlation between the values of the other polyphenols.

The investigated polyphenols could not be detected in native grape seeds by HPLC (data not shown).

### 3.2. Total Antioxidant Capacity

The TAC was determined by three different methods (FCR, FRAP and TEAC) and similar results were found. The highest TAC was found in PN samples compared to BP, S and CS samples with significantly lower antioxidant activities both in native grape seeds and grape pomace (Table 2). In native seeds CS, and in pomace samples BP and S showed the lowest antioxidant capacity. Grape seed pomace samples showed significantly higher TAC than native seeds in all investigated grape varieties measured with three different methods (*p* < 0.001, data not shown)

### 3.3. Fatty Acid Composition

The fatty acid composition and oil content of grape seed pomace is shown in Table 3. The highest oil content was found in the PN and CS pomace seeds followed by S seeds, while the lowest levels were detected in BP samples.

Regarding the SFA composition of grape seed pomace samples, C16:0 (palmitic acid) and C18:0 (stearic acid) were detected in the highest values. The highest level of C16:0 was found in the BP samples, while S samples showed higher C18:0 values than the other investigated grape varieties.

OA was the most abundant monounsaturated fatty acid in grape seed pomace. The highest concentration of OA was found in PN pomace samples, followed by CS, while S and BP samples contained significantly lower levels of OA.

Among the identified polyunsaturated fatty acids, LA was present in the highest concentration in S and ALA in BP grape pomace seeds.

### 3.4. Correlations between Polyphenols and Total Antioxidant Capacities

We found significant positive correlations between resveratrol values and total antioxidant capacities: FCR (Pearson correlation coefficient: +0.935; *p* < 0.000), FRAP (Pearson correlation coefficient: +0.909; *p* < 0.000) and TEAC (Pearson correlation coefficient: +0.900; *p* < 0.000). In contrast, there were no correlations between the antioxidant capacities and the values of the two other investigated polyphenols, rutin and quercetin (data not presented).

### 3.5. Correlations between Polyphenols, Total Antioxidant Capacities and Fatty Acids

The oil content of grape pomace seeds correlated significantly and positively with the resveratrol concentrations and total antioxidant capacities measured with three different methods (Table 4). A similar relationship was found considering the OA, and C20:1n-9 content of the samples. The saturated fatty acid C16:0 showed positive but no significant correlation with the measured resveratrol values. The total antioxidant capacities (FCR, FRAP, TEAC) were in a significant direct proportion with C16:0 concentrations. In contrast, the rutin content of grape pomace samples showed a significant inverse correlation considering the oil content as well as the C16:0, C18:0, OA and C20:1n-9 content of the samples.

**Figure 1:**
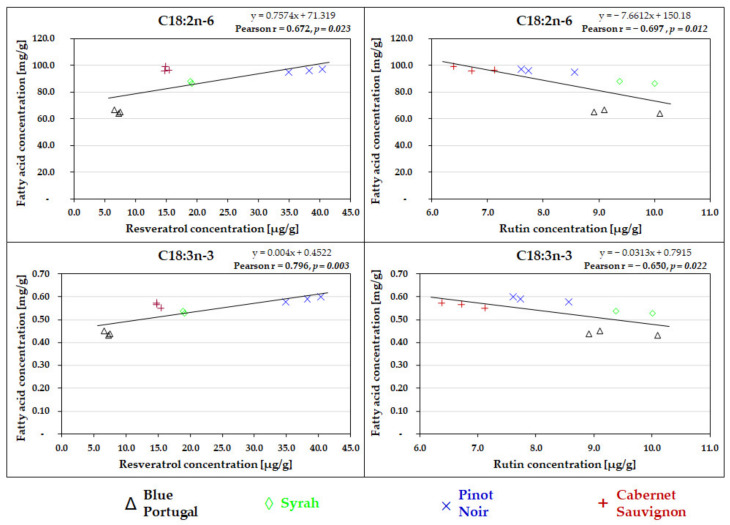
Correlations between the essential fatty acids (C18:3n-3 and C18:2n-6) and polyphenols (resveratrol and rutin) in grape seed pomace of four different grape varieties.

Furthermore, significant positive correlations were found between essential fatty acids (ALA and LA) and the resveratrol concentrations of grape seed pomace samples (Figure 1) ALA and total antioxidant capacities: FCR (Pearson correlation coefficient: +0.705; *p* < 0.05), FRAP (Pearson correlation coefficient: +0.669; *p* < 0.05) and TEAC (Pearson correlation coefficient: +0.676; *p* < 0.05) showed significant positive correlations as well. In contrast, rutin values were in significant inverse proportion with the LA and ALA content of the samples (Figure 1). No significant correlation was found between the antioxidant capacities and LA values; however, there was a trend of positive correlation between LA and FCR (Pearson correlation coefficient: +0.574; *p* = 0.051). There was no significant relationship between fatty acid and quercetin values (data not shown).

## 4. Discussion

In this study, the total antioxidant capacity, polyphenol and oil content as well as the fatty acid composition of both native and fermented grape seeds in four Hungarian widely cultivated red grape varieties were investigated.

The TAC values were higher in all four analyzed grape seed pomace samples than in native grape seeds. We found only one similar measurement: in an Argentine study [40], the antioxidant capacities of whole grape and grape pomace (skin and seeds) extracts in three different varieties were compared. According to these measurements, S and Merlot pomace extracts showed higher antioxidant activities than whole grape extracts in contrast with the CS samples. In whole grape extracts, CS, while in pomace samples, S showed the highest antioxidant capacity.

The TAC measured with three different methods was the highest both in fermented and native seeds of PN variety. Interestingly, CS seeds showed the second-highest antioxidant activity among fermented samples while the lowest values were detected in the unfermented seeds of CS. According to Chambre et al. [41] the antioxidant capacity values of PN seed pomace samples were also higher than that of CS samples.

Similarly to our findings, other studies described that the concentrations of individual flavonols and stilbenes, such as quercetin, rutin and resveratrol, vary greatly in the pomace of different grape varieties [15,25,40].

Numerous studies found strong positive correlation between the antioxidant capacity and the total phenolic content of various grape seeds [1,9,16,42,43,44,45,46,47,48]. Although the total polyphenol content was not determined in our study, a similar significant positive correlation was identified between antioxidant capacities and resveratrol values.

Based on our measurements, the investigated polyphenols could not be detected in native seeds. Similarly, rutin and resveratrol could not be identified in native grape seeds only in grape peel in a Chinese study [47]. Guchu et al. [49] could not detect quercetin either in the unfermented or in fermented grape seed samples. The stilbene content of grape seeds in the different ripening stages of grapes was analyzed by Dudoit et al. [50]. Based on this study, resveratrol could not be detected in the seeds, but it was present in the skin of grapes. Similar results were found by other researchers regarding to the resveratrol content of native seeds in different grape varieties [51,52]. Furthermore, resveratrol [15,25,53], rutin [25,53] and quercetin [25,40] were identified from grape seed pomace samples by other research groups as well. However, some of the previous studies showed conflicting results about the polyphenol content of native grape seeds and pomace. Farhadi et al. [51] investigated six red grape varieties and found low levels of rutin and quercetin in native seed samples. Furthermore, in a Turkish study [54], high levels of rutin, quercetin and resveratrol were found in native grape seeds. Somkuvar et al. [55] could also detect all of the three investigated polyphenols in the native seeds of CS and S red grape varieties. In a study in Brazil [56], neither rutin nor quercetin derivatives could be detected in pomace seed extracts of CS samples.

Considering the oil content of the analyzed grape pomace seeds, the highest values were measured in PN seed samples, followed by S seeds, while the oil content of BP samples was the lowest (Table 3). However, in a Canadian study [23], CS grape pomace seeds showed the highest (11.17 ± 0.05 wt%) and PN samples (9.83 ± 0.05 wt%) the lowest oil content, and the values of S samples were between these two concentrations (10.10 ± 0.10 wt%). Compared to our results, Brazilian CS grape pomace seed samples show lower (4.83 ± 0.11 wt%) [25], while Chinese CS samples higher oil content (14.45 ± 0.03 wt%) [19].

The most abundant fatty acids in grape pomace seed oil are OA and LA. Among the investigated grape pomaces, PN samples had the highest OA content, followed by BP, CS and S. In contrast, one of the previous studies described higher OA content in S grape pomace samples (16.61 wt%) than in PN (15.00 wt%) and CS (12.63 wt%) seeds [23]. Riberio et al. found significantly lower levels of OA (10.01 ± 0.69 wt%) in CS pomace samples [25]. Regarding to our measurements, the highest LA concentrations were detected in S and CS pomace seed samples, while PN and BP seeds contained lower levels of LA. Wen et al. found similar LA concentrations (73.39 ± 0.08 wt%) in CS grape seed pomace [19]. In contrast, Ribeiro et al. found LA in significantly lower amounts (54.58 ± 2.78 wt%) in CS pomace [25]. The results of Beveridge et al. about the LA values of CS (72.77 wt%) and PN (70.00 wt%) grape seed pomace samples correspond well with our findings; however, they detected lower concentrations of LA in S (68.00 wt%) seed samples [23].

Many studies have investigated both the fatty acid composition and polyphenol content of different grape varieties [20,21,43,57,58,59,60], but to the best of our knowledge, this is the first study investigating the relationship between these values. In this study, significant positive correlations were found between the concentration of resveratrol and the oil, OA, C20:1n-9, LA and ALA content of the pomace seed samples. This direct proportion suggests a potential connection between the concentrations and beneficial physiological effects of these molecules. PUFAs may decrease the risk of several diseases, such as dyslipidemia [61], type 2 diabetes mellitus [62], cardiovascular disease [63,64,65], atherosclerosis and metabolic syndrome [66,67]. Resveratrol may also exert beneficial effects in cardiovascular diseases because of its blood pressure lowering [68], anti-inflammatory [69] and NO production-enhancing effects [70]. However, the rutin concentration of the pomace samples correlated significantly and negatively to their OA, C20:1n-9, LA and ALA content. The TAC values of pomace samples correlated significantly and directly to their oil, C16:0, OA, C20:1n-9 and ALA content.

## 5. Conclusions

According to our measurements, the TAC values of grape pomace seeds are significantly higher than that of native grape seeds independent of grape variety. The distinct difference can be explained by their polyphenol contents. The absence of resveratrol, rutin and quercetin in native grape seed samples suggests that the process of fermentation may exert a strong impact on the polyphenol content of grape seeds. Polyphenols present in grape skin, pulp and stem are presumably enriched in grape seeds during fermentation. In addition, a significant positive correlation was found between the resveratrol concentration and the OA, C20:1n-9, LA and ALA content of grape seed pomace samples. Furthermore, a significant inverse proportion was found between rutin values and the concentration of fatty acids in pomace seed samples. Besides, no relationship was found between the quercetin content and the fatty acid composition of grape pomace samples. According to our findings, grape pomace seems a more valuable source of raw material used in the production of modern nutraceuticals due to its higher polyphenol content and stronger antioxidant activity.

## Figures and Tables

**Table 1:** Polyphenol content (mg/kg dry weight) of grape seed pomace of four different grape varieties harvested in the Villány wine region.

	Blue Portugal	Syrah	Pinot Noir	Cabernet Sauvignon
resveratrol (mg/kg)	7.11 ± 0.44 ^ABC^	19.02 ± 0.17 ^ADE^	37.93 ± 2.78 ^BDF^	15.04 ± 0.41 ^CEF^
rutin (mg/kg)	9.37 ± 0.64 ^aA^	8.81 ± 1.56	7.98 ± 0.52 ^ab^	6.74 ± 0.37 ^Ab^
quercetin (mg/kg)	34.17 ± 2.35 ^A^	38.52 ± 1.11 ^Ba^	33.09 ± 0.88 ^Ca^	15.69 ± 0.80 ^ABC^

Means in a row sharing common Roman superscript denote significant differences between the grape varieties: ^a,b^ *p* < 0.05; ^A,B,C,D,E,F^: *p* ≤ 0.001.

**Table 2:** Total antioxidant capacity (FCR, FRAP, TEAC) of native grape seed and grape pomace of four different grape varieties harvested in the Villány wine region.

mg ASA in mg Dry Seed; Mean ± SD		Blue Portugal	Syrah	Pinot Noir	Cabernet Sauvignon
**FCR**	**Native**	0.021 ± 0.001 ^aAB^	0.018 ± 0.000 ^aCD^	0.027 ± 0.000 ^ACE^	0.010 ± 0.000 ^BDE^
	**Pomace**	0.041 ± 0.001 ^AB^	0.041 ± 0.001)^CD^	0.063 ± 0.000 ^ACE^	0.046 ± 0.000 ^BDE^
**FRAP**	**Native**	0.052 ± 0.001 ^AaB^	0.052 ± 0.001 ^ACD^	0.064 ± 0.002 ^aCE^	0.025 ± 0.001 ^BDE^
	**Pomace**	0.078 ± 0.001 ^Aa^	0.077 ± 0.002 ^Bb^	0.116 ± 0.004 ^ABC^	0.085 ± 0.001 ^abC^
**TEAC**	**Native**	0.062 ± 0.002 ^aAB^	0.056 ± 0.002 ^aCD^	0.083 ± 0.001 ^ACE^	0.028 ± 0.001 ^BDE^
	**Pomace**	0.096 ± 0.002 ^Aa^	0.094 ± 0.002 ^Bb^	0.132 ± 0.001 ^ABC^	0.104 ± 0.002 ^abC^

FCR: Folin–Ciocalteu reagent, FRAP: ferric-reducing antioxidant potential, TEAC: Trolox equivalent antioxidant activity. The antioxidant capacities (FCR, FRAP, TEAC) of the different grape varieties (native and pomace) were compared with ANOVA and independent *T*-test. Means in a row sharing common Roman superscript denote significant differences between the grape varieties: ^a,b^ *p* < 0.05; ^A,B,C,D,E^: *p* ≤ 0.001.

**Table 3:** Oil content (g oil/100 g dried seed) and fatty acid composition (wt%, weight percent of total fatty acids) of grape seed pomace of four different grape varieties harvested in the Villány wine region.

	Blue Portugal	Syrah	Pinot Noir	Cabernet Sauvignon
Oil content	9.54 ± 0.21 ^ABC^	12.13 ± 0.14 ^ADa^	13.91 ± 0.21 ^BD^	13.51 ± 0.26 ^Ca^
Saturated fatty acids (SFA)				
C14:0	0.13 ± 0.01 ^ab^	0.10 ± 0.01 ^a^	0.11 ± 0.01 ^c^	0.09 ± 0.01 ^bc^
C15:0	0.02 ± 0.0	0.01 ± 0.00 ^a^	0.02 ± 0.00 ^Aa^	0.01 ± 0.00 ^A^
C16:0	9.68 ± 0.05 ^ABC^	8.05 ± 0.05 ^Aa^	8.03 ± 0.11 ^Bb^	8.34 ± 0.04 ^Cab^
C17:0	0.07 ± 0.00 ^a^	0.07 ± 0.00 ^b^	0.07 ± 0.00 ^abc^	0.06 ± 0.00 ^c^
C18:0	4.12 ± 0.02 ^ABC^	4.55 ± 0.01 ^ADE^	3.85 ± 0.00 ^BDF^	4.45 ± 0.02 ^CEF^
C20:0	0.17 ± 0.00 ^AaB^	0.20 ± 0.00 ^AC^	0.16 ± 0.00 ^aCD^	0.20 ± 0.01 ^BD^
C22:0	0.06 ± 0.01 ^a^	0.07 ± 0.00	0.07 ± 0.00 ^ab^	0.06 ± 0.01 ^b^
C24:0	0.04 ± 0.00 ^ABa^	0.03 ± 0.00 ^A^	0.03 ± 0.00 ^Bb^	0.04 ± 0.00 ^ab^
Monounsaturated fatty acids (MUFA)				
C16:1n-7	0.20 ± 0.00 ^ABC^	0.09 ± 0.00 ^AD^	0.16 ± 0.00 ^BDE^	0.09 ± 0.00 ^CE^
C18:1n-9	15.40 ± 0.03 ^ABC^	13.37 ± 0.01 ^AD^	16.80 ± 0.15 ^BDE^	13.53 ± 0.13 ^CE^
C18:1n-7	0.82 ± 0.01 ^AaB^	0.65 ± 0.02 ^AC^	0.81 ± 0.00 ^aCD^	0.66 ± 0.02 ^BD^
C20:1n-9	0.14 ± 0.00 ^Aa^	0.16 ± 0.00 ^A^	0.17 ± 0.01 ^a^	0.16 ± 0.01
Polyunsaturated fatty acids (PUFA)				
C18:2n-6	68.43 ± 0.05 ^AaB^	71.95 ± 0.07 ^ACb^	69.01 ± 0.24 ^aCD^	71.61 ± 0.12 ^BbD^
C18:3n-3	0.46 ± 0.00 ^ABC^	0.44 ± 0.00 ^Aab^	0.42 ± 0.00 ^Ba^	0.42 ± 0.01 ^Cb^

Means in a row sharing common Roman superscript denote significant differences between the grape varieties: ^a,b,c^: *p* < 0.05; ^A,B,C,D,E^: *p* ≤ 0.001.

**Table 4:** Pearson’s correlation coefficients of total antioxidant capacities, oil content, fatty acid and polyphenol concentrations in grape seed pomace samples.

	Resveratrol		Rutin		FCR		FRAP		TEAC	
	Pearson	Sig	Pearson	Sig	Pearson	Sig	Pearson	Sig	Pearson	Sig
Oil Content	**0.747**	**0.008**	**−0.686**	**0.014**	**0.678**	**0.015**	**0.643**	**0.024**	**0.654**	**0.021**
C16:0	0.555	0.077	**−0.864**	**0.001**	**0.691**	**0.013**	**0.643**	**0.024**	**0.682**	**0.015**
C18:0	0.273	0.417	**−0.664**	**0.026**	0.262	0.411	0.224	0.484	0.233	0.466
C18:1n-9	**0.800**	**0.003**	**−0.673**	**0.023**	**0.972**	**0.000**	**0.950**	**0.000**	**0.959**	**0.000**
C20:1n-9	**0.691**	**0.019**	**−0.700**	**0.016**	**0.695**	**0.012**	**0.655**	**0.021**	**0.659**	**0.020**

Bold numbers denote significant correlations.

## Data Availability

The data presented in this study are available on request from the corresponding author.
